# Female Medical Education

**Published:** 1870-02

**Authors:** 


Female Medical Education.—The Faculty of the University of Edinburg has completed the arrangements for enabling females to study medicine. Separate classes for males and females have been formed, and five women have already presented themselves for examination for matriculation. A female medical society, under the presidency of the Earl of Shaftesbury, has been established in London, with the objects of providing educated women with proper facilities for learning the necessary branches of medicine, and of promoting the employment of female physicians for the treatment of the diseases of women and children.

